# Unpredictable and Poisson Stable Oscillations of Inertial Neural Networks with Generalized Piecewise Constant Argument

**DOI:** 10.3390/e25040620

**Published:** 2023-04-06

**Authors:** Marat Akhmet, Madina Tleubergenova, Zakhira Nugayeva

**Affiliations:** 1 Department of Mathematics, Middle East Technical University, Ankara 06800, Turkey; 2 Department of Mathematics, Aktobe Regional University, Aktobe 030000, Kazakhstan; 3 Institute of Information and Computational Technologies, Almaty 050000, Kazakhstan

**Keywords:** inertial neural networks, generalized piecewise constant argument, unpredictable oscillations, Poisson stable oscillations, unpredictable input–outputs, Poisson triple, Poincaré chaos, exponential stability

## Abstract

A new model of inertial neural networks with a generalized piecewise constant argument as well as unpredictable inputs is proposed. The model is inspired by unpredictable perturbations, which allow to study the distribution of chaotic signals in neural networks. The existence and exponential stability of unique unpredictable and Poisson stable motions of the neural networks are proved. Due to the generalized piecewise constant argument, solutions are continuous functions with discontinuous derivatives, and, accordingly, Poisson stability and unpredictability are studied by considering the characteristics of continuity intervals. That is, the piecewise constant argument requires a specific component, the Poisson triple. The *B*-topology is used for the analysis of Poisson stability for the discontinuous functions. The results are demonstrated by examples and simulations.

## 1. Introduction

Recently, digitalization and artificial intelligence have been actively introduced into many spheres of life, and neural networks, due to the unique structure, and high efficiency of information processing, have become the main tool for their implementation. Basic neural network models, such as cellular neural networks [1], bidirectional associative memory neural networks [2], Hopfield neural networks [3] and shunting inhibitory cellular neural networks [4] are described using first-order differential equations. However, it was found that these mentioned neural networks cannot effectively model the mechanism of squid semicircular canal and synapse [5]. To solve such an actual problem, Babcock and Westervelt [6,7] introduced neural networks, known today as an inertial neural networks (INNs). The standard mathematical model of the INNs can be described by the second-order derivative and differs from the Hopfield neural networks by the inertial term. The inclusion of inertia in the neural network model opened up new directions in the field biology, engineering technology and information system [8,9].

The original inertial neural networks was considered in [10],

(1) $$u_{i}^{\prime \prime }\left(t\right)=-a_{i}u_{i}^{\prime }\left(t\right)-b_{i}u_{i}\left(t\right)+\sum_{j=1}^{m}c_{ij}f_{j}\left(u_{j}\left(t\right)\right)+I_{i}\left(t\right),$$

where $t,\;u_{i}\in R,\;i=1,2,\ldots m$, the second derivative of $u_{i}\left(t\right)$, is called an inertial term of system (1); $u_{i}\left(t\right)$ is the state variable of the *i* th neuron at time *t*; $a_{i}>0$ is the damping coefficient; $b_{i}>0$ is the rate with which the neurons self-regulate or reset their potential when isolated from other networks and inputs; the constant $c_{ij}$ is the synaptic connection weight of the neuron *j* on the neuron *i*; $f_{j}$ is the activation function of incoming potentials of the neuron *j* at time *t*; and $I_{i}\left(t\right)=I_{i}$ is the external input of network to the *i*th neuron.

In recent years, by using the reduced-order transformation, numerous papers have been written on the stability and synchronization of the following delayed INNs and its generalizations [11,12,13],

(2) $$u_{i}^{\prime \prime }\left(t\right)=-a_{i}u_{i}^{\prime }\left(t\right)-b_{i}u_{i}\left(t\right)+\sum_{j=1}^{m}c_{ij}f_{j}\left(u_{j}\left(t\right)\right)+\sum_{j=1}^{m}d_{ij}f_{j}\left(u_{j}\left(t-\tau_{ij}\right)\right)+I_{i}\left(t\right),$$

where time delay $\tau_{ij}\geq 0$ is constant, $d_{ij}$ is the synaptic connection weight of the neuron *j* on the neuron *i* with delay, and the external input $I_{i}\left(t\right)$ for all i=1,2,…m is continuous functions.

In [14], the inertial neural network (1) was modified by adding mixed delays,

(3) $$\begin{array}{ccc} u_{i}^{\prime \prime }\left(t\right)= & & -a_{i}u_{i}^{\prime }\left(t\right)-b_{i}u_{i}\left(t\right)+\sum_{j=1}^{m}c_{ij}f_{j}\left(u_{j}\left(t\right)\right)+\sum_{j=1}^{m}d_{ij}f_{j}\left(u_{j}\left(t-\tau_{j}\left(t\right)\right)\right)+\\ & & +\sum_{j=1}^{m}w_{ij}\int_{t-k_{j}\left(t\right)}^{t}f_{j}\left(u_{j}\left(s\right)\right)ds+I_{i}\left(t\right), \end{array}$$

where $c_{ij}$, $d_{ij}$ and $w_{ij}$ are the synaptic connection weights, which are related to the neurons without delays, with discrete delay and distributive delay, separately. $\tau_{j}$ is the discrete delay; meanwhile, $k_{j}\left(t\right)$ is the distributed delay, which satisfies $0\leq \tau_{j}\leq \tau$, $0\leq k_{j}\left(t\right)\leq k$, where τ and *k* are constants. The external input $I_{i}\left(t\right)$ for all i=1,2,…m, is continuous functions.

The latest results are related to consideration of time-varying velocities, weights of connections and external inputs as continuous periodic, almost periodic functions depending on a time variable. These results have been effectively applied to numerous fields, such as the stabilization of periodic and almost anti-periodic motions, and control, as well as synchronization [15,16,17,18,19,20].

In recent years, scientists have begun to study the distribution of recurrent and chaotic signals in the network, taking Poisson stable [21,22,23,24,25] and unpredictable functions [26] as external inputs. We note that the Poisson stable functions are a complex and general case of the class of recurrent functions known to us as almost periodic, quasi-periodic and periodic functions [27]. The concept of the unpredictability was introduced in 2016 [26]. Trajectories of the dynamics are of Poincaré chaos, and this provides strong argument for the research, considering the theoretical and application merits of chaos. Unpredictable functions differ from the Poisson stable functions by the separation property [27,28]. Thus, unpredictable motions make a subclass of Poisson stable ones, but as the studies demonstrate, all examples of concrete Poisson stable functions are unpredictable. That is, the unpredictability may be a more constructive phenomenon than Poisson stability [27,28]. Nevertheless, to provide complete information of the results, we prove two main assertions on both unpredictable and Poisson stable motions in the neural networks. Poisson stability is proved by *the method of included intervals*, which was introduced and developed in [27,28,29]. Note that, currently, this method remains the main way to prove convergence, due to its efficiency in theory differential equations.

For the first time, unpredictable motions were investigated for inertial neural networks in [29], that is, unpredictable functions were used as input data in system (1). The article considers the case when the reduced-order transformation formula includes all parameters, unlike the articles [10,14,15,18,19]. For example, if in the article [19] strict restrictions are imposed on the coefficients in the main transformation formula, then in [29], for a broader study, the positive coefficients are considered. The presence of such coefficients in the transformation [29] give advantages in a detailed study of the behavior of the solution.

The present and potential contributions as well as the novelties of the paper can be highlighted as follows:

- In this article, the existence, uniqueness and exponential stability of discontinuous Poisson stable and unpredictable motions of inertial neural networks with a generalized piecewise constant argument are studied.
- It is the first time in the literature that an inertial neural network with a generalized piecewise constant argument, combining delayed and advanced arguments, is investigated.
- The generalized piecewise constant argument function in the neural network model is given in the form of a discontinuous stable Poisson function, which, in turn, is also a novelty of the work. The function is described by special sequences that are connected by a newly introduced relation, that is, a Poisson triple of sequences.
- Poisson stability of discontinuous functions is studied on the basis of the *B*-topology and by the method of included intervals.
- In the future, the included intervals method based on *B*-topology can be used for neural networks with both impulsive and discontinuous functional dependence on time.

## 2. Preliminaries

Our goal is to find unpredictable and Poisson stable motions of inertial neural network with a generalized piecewise constant argument. The presence of a piecewise constant argument requires the study of unpredictable and Poisson stable oscillations on each continuous interval. That is, we need to enter an unpredictable function and a piecewise constant argument function. For that, in this part of the paper, we introduce special time sequences and describe the generalized piecewise constant argument.

Denote by N,R and Z the sets of natural and real numbers, and integers, respectively. Let ∥·∥ be the Euclidean norm in $R^{m},m\in N$.

Let us commence with preliminary concepts and give the basic definitions of the Poisson stable sequence and function, and the unpredictable function.

**Definition** **1** ([22]). *A sequence $\kappa_{i},i\in Z$, in R is called Poisson stable, provided that it is bounded and there exists a sequence $l_{n}\to \infty$, n∈N, of positive integers such that $|\kappa_{i+l_{n}}-\kappa_{i}|\to$ as n→∞ on bounded intervals of integers.*

**Definition** **2** ([27]). *A uniformly continuous and bounded function $\phi :R\to R^{m}$ is unpredictable if there exist positive numbers $\epsilon_{0},\delta$ and sequences $t_{n},s_{n}$ both of which diverge to infinity such that $∥\phi \left(t+t_{n}\right)-\phi \left(t\right)∥\to o$ as n→∞ uniformly on compact subsets of R and $∥\phi \left(t+t_{n}\right)-\phi \left(t\right)∥\geq \epsilon_{0}$ for each $t\in [s_{n}-\delta ,s_{n}+\delta ]$ and n∈N.*

**Definition** **3** ([22]). *A continuous and bounded function $\phi \left(t\right):R\to R^{n}$ is said to be Poisson stable if there exists a sequence $t_{n},$$t_{n}\to \infty$ as n→∞, such that $∥\phi \left(t+t_{n}\right)-\phi \left(t\right)∥\to 0$ uniformly on compact subsets of R*.

The sequence $t_{n}$ in the last definitions is called the *Poisson or convergence sequence* and the divergence estimated by $\epsilon_{0}$ is said to be the *separation property*.

In this paper, the main subject for investigation is inertial neural networks with piecewise constant argument. The differential equations with piecewise constant arguments, like any model of the theory of differential equations, arose due to the need to apply them in real life. They are important mathematical models that are used in the study of biological, biomedical processes, control theory, stabilization and neural networks [30,31,32]. For example, in control theory, the following process can be investigated using differential equations with a piecewise constant argument: a state and control constraints, a plant under control, as well as the performance index to be minimized are defined in continuous time, while the manipulated variables are permitted to change at fixed and uniformly distributed sampling times [33].

In general, differential equations with a piecewise constant argument have the form,

$$x^{\prime }=f\left(t,x\left(t\right),x\left(\gamma \left(t\right)\right)\right),$$

where γ(t) is a generalized piecewise constant argument function. It should be noted that although the function γ(t) is discontinuous, the solution of the differential equation will be continuous functions. A full description of this discontinuous function γ(t) can be found in the book [33].

The system that we will consider has the following form:

(4) $$u_{i}^{\prime \prime }\left(t\right)=-a_{i}u_{i}^{\prime }\left(t\right)-b_{i}u_{i}\left(t\right)+\sum_{j=1}^{m}c_{ij}f_{j}\left(u_{j}\left(t\right)\right)+\sum_{j=1}^{m}d_{ij}g_{j}\left(u_{j}\left(\gamma \left(t\right)\right)\right)+h_{i}\left(t\right),$$

where $t,\;u_{i}\in R,\;i=1,2,\ldots m,\;\gamma \left(t\right)$ is a generalized piecewise constant argument function. Similar to coefficients $c_{ij}$ of system (1), the constant $d_{ij}$ is the synaptic connection weight of the neuron *j* on the neuron i, and $g_{j}$ is the activation function of incoming potentials of the neuron *j* at time *t*, respectively. Moreover, inputs $h_{i}\left(t\right)$ in system (4) are unpredictable functions.

In what follows, we assume that the activations $f_{j},g_{j}:R\to R,\;\;j=1,2,\cdots ,m,$ are continuous functions, and the parameters $c_{ij}$ and $d_{ij}$ are real numbers.

### 2.1. Poisson Sequences

Fix the sequences of real numbers $t_{n},\theta_{k},\;\xi_{k},\;n\in N,k\in Z,$ which are strictly increasing with respect to the index. Sequences $\theta_{k},\;\xi_{k},\;k\in Z,$ are unbounded in both directions. In what follows, we call them *Poisson sequences*.

We provide the description of the Poisson couple and Poisson triple in the following definitions.

**Definition** **4.** *A couple $\left(t_{n},\theta_{k}\right)$ of Poisson sequences $t_{n},\theta_{k}$, n∈N,k∈Z, is called a Poisson couple if there exists a sequence $l_{n}$, n∈N of integers, satisfying $l_{n}\to \infty$, as n→∞ such that*

(5) $$\theta_{k+l_{n}}-t_{n}-\theta_{k}\to 0\;as\;n\to \infty ,$$

*uniformly on each bounded interval of integers k.*

**Definition** **5.** *A triple $\left(t_{n},\theta_{k},\xi_{k}\right)$ of the sequences $t_{n},\theta_{k},\xi_{k}$, n∈N,k∈Z, is called Poisson triple, if there exists a sequence $l_{n}$, n∈N of integers, satisfying $l_{n}\to \infty$ as n→∞ such that the condition (5) is fulfilled, and*

(6) $$\xi_{k+l_{n}}-t_{n}-\xi_{k}\to 0\;as\;n\to \infty ,$$

*uniformly on each bounded interval of integers k.*

By comparing the Definitions 4 and 5, one can formulate the following definition, which is equivalent to Definition 5:

**Definition** **6.** *A triple $\left(t_{n},\theta_{k},\xi_{k}\right)$ of the sequences $t_{n},\theta_{k},\xi_{k}$, n∈N,k∈Z, is called a Poisson triple, if the couples $\left(t_{n},\theta_{k}\right)$, $\left(t_{n},\xi_{k}\right)$ are separately Poisson couples with the common convergence sequence $l_{n}$.*

**Definition** **7** ([34]). *A sequence $\tau_{k}$, k∈Z, is said to be with the (w,p)—property if there exist positive real number w and integer p, which satisfy $\tau_{k+p}-\tau_{k}=w$ for all k∈Z.*

Next, we will consider important properties of sequences that will be used in the study of further theoretical and illustrating the results.

**Lemma** **1.** *Assume that the couple $\left(t_{n},\theta_{k}\right)$ of the sequences $t_{n},\theta_{k}$, n∈N,k∈Z, satisfies the following conditions:*

*(i)*  *$t_{n}=nw$, where w∈R, n∈N;*
*(ii)*  *the sequence $\theta_{k}$ admits the (w,p)− property.*

*Then $\left(t_{n},\theta_{k}\right)$ is a Poisson couple.*

**Proof.**  Since (w,p)− property is true, $\theta_{k+p}=\theta_{k}+w$ for each k∈Z. Taking $l_{n}=np$ for n∈N, we obtain that $\theta_{k+np}=\theta_{k}+nw$. Now, one can easily check that the sequence $\theta_{k+l_{n}}-t_{n}-\theta_{k}$ consists of zeros. Thus, the condition (5) is satisfied on each bounded interval of integers *k*. □

**Lemma** **2.** *Assume that the triple $\left(t_{n},\theta_{k},\xi_{k}\right)$ of the sequences $t_{n},\theta_{k}$, $\xi_{k},n\in N,k\in Z$ consists of the Poisson couple $\left(t_{n},\theta_{k}\right)$ such that*

*(i)*  *$t_{n}=nw$, where w∈R, n∈N,*
*(ii)*  *the sequence $\theta_{k}$ admits the (w,p)− property,*

*and the sequence $\xi_{k}=\theta_{k}+s_{k}$, satisfying the following conditions:*

*(iii)*  *the sequence $s_{k}$ is p—periodic.*
*(iv)*  *for all k∈Z it is true that $0\leq s_{k}<\max_{k}\left(\theta_{k+1}-\theta_{k}\right)$.*

*Then $\left(t_{n},\theta_{k},\xi_{k}\right)$ is a Poisson triple.*

**Proof.**  According to Lemma 1, $\left(t_{n},\theta_{k}\right)$ is a Poisson couple. So, it remains to check the validity of $\xi_{k+l_{n}}-t_{n}-\xi_{k}\to 0$ as n→∞ uniformly on each bounded interval of integers *k*. By using the periodicity property, $s_{k+np}=s_{k},$ and for all *k*, one can see that the sequence $\xi_{k+l_{n}}-t_{n}-\xi_{k}$ consists of zeros. That is, (6) is valid on each bounded interval of integers *k*. □

As an example of a Poisson triple, one can consider $\left(t_{n},\theta_{k},\xi_{k}\right)$, where $t_{n}=nw,n\in N,$ and $\theta_{k}=\frac{k}{5}$, $\xi_{k}=\frac{\theta_{k}+\theta_{k+1}}{2}=\frac{2k+1}{10},\;k\in Z$. One can verify that the sequence $\theta_{k}$ satisfies $(\frac{1}{5},1)—$periodic property and $\xi_{k}=\theta_{k}+s_{k}$, where $s_{k}=\frac{1}{10}$. That is, the triple satisfies the conditions of Lemma 2.

### 2.2. Description of the Generalized Piecewise Constant Argument

Let us determine the argument function in (4). In this paper, it is assumed that $\gamma \left(t\right)=\xi_{k}$ if $\theta_{k}\leq t<\theta_{k+1}$, k∈Z, and the function is defined on the whole real line with two Poisson sequences $\theta_{k},\;\xi_{k},\;k\in Z$ such that $\underline{\theta }\leq \theta_{k+1}-\theta_{k}\leq \bar{\theta }$ for some positive numbers $\underline{\theta }$, $\bar{\theta },$ and all integers k.

Additionally to the sequences $\theta_{k},\;\xi_{k}$, we fix a sequence $t_{n}$, n∈Z, such that $\left(t_{n},\theta_{k},\xi_{k}\right)$, is a Poisson triple in the sense of Definition 5. Consider the function $\gamma (t+t_{n})$ for a fixed n∈Z. It is possible to show that $\gamma \left(t+t_{n}\right)=\xi_{k+l_{n}}$ if $\theta_{k}^{\prime }\leq t<\theta_{k+1}^{\prime }$, where $\theta_{k}^{\prime }=\theta_{k+l_{n}}-t_{n}$, k∈Z.

Next, we shall show that the discontinuous argument function admits properties, which are analogues of the Poisson stability.

Fix a bounded interval [a,b] with b>a, and an arbitrary positive number ϵ such that $2\epsilon <\underline{\theta }$ on this interval. We assume without loss of generality that $\theta_{k}\leq \theta_{k+l_{n}}-t_{n}$ and consider discontinuity moments $\theta_{k}$, k=l+1,l+2,…,l+p−1, of the interval [a,b] such that

$$\theta_{l}\leq a<\theta_{l+1}<\theta_{l+2}<\cdots <\theta_{l+p-1}<b\leq \theta_{l+p}.$$

We show that for sufficiently large *n*, it is true that

(7) $$|\theta_{k}^{\prime }-\theta_{k}|<\epsilon$$

for all k=l,l+1,…,l+p, and

(8) $$|\gamma \left(t+t_{n}\right)-\gamma \left(t\right)|<\epsilon$$

for each t∈[a,b], except those between $\theta_{k}$ and $\theta_{k}^{\prime }$ for each *k*.

Let us fix *k*, k=l,l+1,…,l+p, and for fixed *k*, we have that $\gamma \left(t\right)=\xi_{k}$, for $t\in [\theta_{k},\theta_{k+1})$ and $\gamma \left(t+t_{n}\right)=\xi_{k+l_{n}}$, $t\in [\theta_{k}^{\prime },\theta_{k+1}^{\prime })$. Thus, for sufficiently large *n*, the interval $\left(\theta_{k}^{\prime },\theta_{k+1}\right)$ is non-empty. According to (5), condition (7) is valid. Moreover, from condition (6), it is implied that for sufficiently large n,

(9) $$|\gamma \left(t+t_{n}\right)-\gamma \left(t\right)\left|=\right|\xi_{k+l_{n}}-\xi_{k}|<\epsilon$$

for $t\in [\theta_{k}^{\prime },\theta_{k+1})$. Thus, inequalities (7) and (8) are approved.

If conditions (7) and (8) are valid for arbitrary ϵ, then the piecewise constant function $\gamma (t+t_{n})$ converges to the function γ(t) on the bounded interval in *B*-topology [34]. That is, γ(t) is a *discontinuous Poisson stable function*.

It should be noted that in this article, all the coefficients in (4) are constant. If one wants to consider the coefficients $a_{i},b_{i},c_{ij},d_{ij}$ variable, they would be periodic or even unpredictable. That is, we need a special *kappa property* [35], which establishes a correspondence between periodicity and the unpredictability. The existence of such factors should be due to the higher possibility of the selection of the triple $t_{n},\theta_{k},\xi_{k}$, when they satisfy (w,p)—property [34], and in addition, the kappa property must be fulfilled [35,36,37,38]. In this paper, we utilize a stronger state when this triple is a Poisson triple, which is more comfortable in applications. This is why, in order not to weaken circumstances, we agree that the coefficients are constants.

Throughout the article, the components of the generalized piecewise constant argument γ(t) in the system (4) are connected by the Poisson triple $\left(t_{n},\theta_{k},\xi_{k}\right)$, and they are understood as mentioned in this subsection.

### 2.3. Reduced System

As mentioned in the introduction, we use the following transformation formula [29]:

(10) $$v_{i}\left(t\right)=\alpha_{i}u_{i}^{\prime }\left(t\right)+\beta_{i}u_{i}\left(t\right),\;i=1,\cdots ,m,$$

and correspondingly, rewrite the neural network system (4) as

(11) $$\left\{\begin{array}{c} u_{i}^{\prime }\left(t\right)=-\frac{\beta_{i}}{\alpha_{i}}u_{i}\left(t\right)+\frac{1}{\alpha_{i}}v_{i}\left(t\right),\;\;\;\;\;\;\;\;\;i=1,\cdots ,m\\ v_{i}^{\prime }\left(t\right)=-\left(a_{i}-\frac{\beta_{i}}{\alpha_{i}}\right)v_{i}\left(t\right)-\left(\alpha_{i}b_{i}-\beta_{i}\left(a_{i}-\frac{\beta_{i}}{\alpha_{i}}\right)\right)u_{i}\left(t\right)\\ \;+\alpha_{i}\sum_{j=1}^{m}c_{ij}f_{j}\left(u_{j}\left(t\right)\right)+\alpha_{i}\sum_{j=1}^{m}d_{ij}g_{j}\left(u_{j}\left(\gamma \left(t\right)\right)\right)+\alpha_{i}h_{i}\left(t\right),\;i=1,\cdots ,m. \end{array}\right.$$

If we take into account the contents of articles [10,14,15,18,19], the variable transformation formula considers the case when $\alpha_{i}=1.$ The presence of the two parameters $\alpha_{i},\beta_{i}$ makes the results more general.

### 2.4. A Space of Functions

Introduce the set $\Sigma_{0}$ of 2m-dimensional vector-functions $\psi \left(t\right)=(\psi_{1}\left(t\right),\psi_{2}\left(t\right),\ldots ,$$\psi_{2m}\left(t\right))$ with the norm ${∥\psi ∥}_{1}=\underset{t\in R}{\sup}∥\psi (t)∥.$ It is assumed that the functions of $\Sigma_{0}$ satisfy the following properties:

(A1) They are Poisson stable functions with the common sequence of convergence $t_{n},n=1,2,\ldots$.
(A2) There exists a number H>0 such that ${∥\psi ∥}_{1}<H$ for all functions.

The following assumptions on the system (4) are required:

(C1) $|f_{i}\left(u\right)-f_{i}\left(v\right)|\leq L_{i}\left|u-v\right|$ and $|g_{i}\left(u\right)-g_{i}\left(v\right)|\leq {\bar{L}}_{i}\left|u-v\right|$ for all $u,v\in R^{m},$ where $L_{i},{\bar{L}}_{i}$ are positive constants for i=1,2,…,m;
(C2) $|h_{i}\left(t\right)|\leq H$, $|f_{i}\left(u\right)|\leq m_{f}$ and $|g_{i}\left(u\right)|\leq m_{g}$, where $m_{f}$, $m_{g}$ are positive numbers for all i=1,2,…,m, |u|<H;
(C3) $a_{i}>\frac{\beta_{i}}{\alpha_{i}}+\alpha_{i},\beta_{i}>\alpha_{i}>1$, i=1,2,…,m;
(C4) $\left(a_{i}-\frac{\beta_{i}}{\alpha_{i}}\right)-\left(\right|\beta_{i}\left(a_{i}-\frac{\beta_{i}}{\alpha_{i}}\right)-\alpha_{i}b_{i}\left|+\alpha_{i}\right)>0$, for each i=1,2,…,m;
(C5) $\frac{\alpha_{i}\left(m_{f}\sum_{j=1}^{m}c_{ij}+m_{g}\sum_{j=1}^{m}d_{ij}\right)}{\left(a_{i}-\frac{\beta_{i}}{\alpha_{i}}\right)-\left(\right|\beta_{i}\left(a_{i}-\frac{\beta_{i}}{\alpha_{i}}\right)-\alpha_{i}b_{i}\left|+\alpha_{i}\right)}<H$, i=1,2,…,m;
(C6) $\frac{1}{\left(a_{i}-\frac{\beta_{i}}{\alpha_{i}}\right)}\left[|\beta_{i}\left(a_{i}-\frac{\beta_{i}}{\alpha_{i}}\right)-\alpha_{i}b_{i}|+\alpha_{i}\left(L_{i}\sum_{j=1}^{m}c_{ij}+{\bar{L}}_{i}\sum_{j=1}^{m}d_{ij}\right)\right]<1$, i=1,2,…,m.

## 3. Main Results

This section of the manuscript concerns the existence and stability of the dynamics, which is discussed in *Preliminaries*, that is, Poisson and unpredictable oscillations of INNs (4). The investigation is fulfilled by considering dynamics of the specific operator Π in the space $\Sigma_{0}.$ We prove the existence of Poisson stable dynamics in neural networks based on the invariance and completeness of the operator in the set. Further, the existence and exponential stability of the unpredictable solutions are confirmed.

**Lemma** **3.** *A couple $u\left(t\right)=(u_{1}\left(t\right),\ldots ,u_{m}\left(t\right))$, and $y\left(t\right)=(y_{1}\left(t\right),\ldots ,y_{m}\left(t\right))$ is a bounded solution of Equation (4) if and only if it is a solution of the following integral equation:*

(12) $$\left\{\begin{array}{c} u_{i}\left(t\right)=\frac{1}{\alpha_{i}}\int_{-\infty }^{t}e^{-\frac{\beta_{i}}{\alpha_{i}}\left(t-s\right)}y_{i}\left(s\right)ds,\\ v_{i}\left(t\right)=\int_{-\infty }^{t}e^{-\left(a_{i}-\frac{\beta_{i}}{\alpha_{i}}\right)\left(t-s\right)}[\left(\beta_{i}\left(a_{i}-\frac{\beta_{i}}{\alpha_{i}}\right)-\alpha_{i}b_{i}\right)u_{i}\left(s\right)\\ \;+\alpha_{i}\sum_{j=1}^{m}c_{ij}f_{j}\left(u_{j}\left(s\right)\right)+\alpha_{i}\sum_{j=1}^{m}d_{ij}g_{j}\left(u_{j}\left(\gamma \left(s\right)\right)\right)+\alpha_{i}h_{i}\left(s\right)]ds, \end{array}\right.$$

*with i=1,⋯,m.*

Define in $\Sigma_{0}$ the operator Π such that $\Pi \psi \left(t\right)=(\Pi_{1}\psi_{1}\left(t\right),\Pi_{2}\psi_{2}\left(t\right),\cdots ,\Pi_{2m}\psi_{2m}\left(t\right))$, where

(13) $$\Pi_{i}\psi_{i}\left(t\right)=\left\{\begin{array}{c} \frac{1}{\alpha_{i}}\int_{-\infty }^{t}e^{-\frac{\beta_{i}}{\alpha_{i}}\left(t-s\right)}\psi_{i+m}\left(s\right)ds,\;\;\;\;\;\;\;i=1,\cdots ,m,\\ \int_{-\infty }^{t}e^{-\left(a_{i-m}-\frac{\beta_{i-m}}{\alpha_{i-m}}\right)\left(t-s\right)}[\left(\beta_{i-m}\left(a_{i-m}-\frac{\beta_{i-m}}{\alpha_{i-m}}\right)-\alpha_{i-m}b_{i-m}\right)\psi_{i-m}\left(s\right)\\ +\alpha_{i-m}\sum_{j=1}^{m}c_{(i-m)j}f_{j}\left(\psi_{j}\left(s\right)\right)+\alpha_{i-m}\sum_{j=1}^{m}d_{(i-m)j}g_{j}\left(\psi_{j}\left(\gamma \left(s\right)\right)\right)\\ +\alpha_{i-m}h_{i-m}\left(s\right)]ds,\;\;\;\;\;\;i=m+1,\cdots ,2m. \end{array}\right.$$

**Lemma** **4.** *
$\Pi \Sigma_{0}\subseteq \Sigma_{0}.$
*

**Proof.**  We have for $\psi_{i}\left(t\right)\in \Sigma_{0}$ and fixed i=1,⋯,m that

$$|\Pi_{i}\psi_{i}\left(t\right)|=\left\{\begin{array}{c} |\frac{1}{\alpha_{i}}\int_{-\infty }^{t}e^{-\frac{\beta_{i}}{\alpha_{i}}\left(t-s\right)}\psi_{i+m}\left(s\right)ds|\leq \frac{1}{\beta_{i}}\left|\psi_{i+m}\left(t\right)\right|\leq \frac{H}{\beta_{i}},\;\;\;i=1,\cdots ,m,\\ |\int_{-\infty }^{t}e^{-\left(a_{i-m}-\frac{\beta_{i-m}}{\alpha_{i-m}}\right)\left(t-s\right)}[\left(\beta_{i-m}\left(a_{i-m}-\frac{\beta_{i-m}}{\alpha_{i-m}}\right)-\alpha_{i-m}b_{i-m}\right)\psi_{i-m}\left(s\right)\\ +\alpha_{i-m}\sum_{j=1}^{m}c_{(i-m)j}f_{j}\left(\psi_{j}\left(s\right)\right)+\alpha_{i-m}\sum_{j=1}^{m}d_{(i-m)j}g_{j}\left(\psi_{j}\left(\gamma \left(s\right)\right)\right)+\alpha_{i-m}\vartheta_{i-m}\left(s\right)]ds|\\ \leq \int_{-\infty }^{t}e^{-\left(a_{i-m}-\frac{\beta_{i-m}}{\alpha_{i-m}}\right)\left(t-s\right)}[|\beta_{i-m}\left(a_{i-m}-\frac{\beta_{i-m}}{\alpha_{i-m}}\right)-\alpha_{i-m}b_{i-m}|H\\ +\alpha_{i-m}\sum_{j=1}^{m}c_{(i-m)j}m_{f}+\alpha_{i-m}\sum_{j=1}^{m}d_{(i-m)j}m_{g}+\alpha_{i-m}H]ds\\ \leq \frac{1}{\left(a_{i-m}-\frac{\beta_{i-m}}{\alpha_{i-m}}\right)}[\left(\beta_{i-m}\left(a_{i-m}-\frac{\beta_{i-m}}{\alpha_{i-m}}\right)-\alpha_{i-m}b_{i-m}\right)|H\\ +\alpha_{i-m}\sum_{j=1}^{m}c_{(i-m)j}m_{f}+\alpha_{i-m}\sum_{j=1}^{m}d_{(i-m)j}m_{g}+\alpha_{i-m}H],\;\;\;i=m+1,\cdots ,2m. \end{array}\right.$$

From the last inequality and conditions (C4) and (C5), we obtain ${\left||\Pi \psi |\right|}_{1}<H$. So, the property (A2) is valid for Πψ.

We continue the proof and show that Πψ satisfies condition (A1) using the method of included intervals [27,28,29]. We need to verify that there exists a sequence $t_{n}$, satisfying $t_{n}\to \infty$, as n→∞ such that for each $\Pi \psi \in \Sigma_{0},$$\Pi \psi \left(t+t_{n}\right)\to \Pi \psi \left(t\right)$ uniformly on each closed and bounded interval of R. Fix an arbitrary number ε>0 and an interval [a,b] with a<b, where a,b∈R. It is enough to show that $||\Pi \psi \left(t+t_{n}\right)-\Pi \psi \left(t\right)\left|\right|<\varepsilon$ for sufficiently large *n* and t∈[a,b]. One can find numbers c<a and ξ>0 in order to fulfill the following inequalities:

(14) $$\frac{2H}{\beta_{i}}e^{-\frac{\beta_{i}}{\alpha_{i}}\left(a-c\right)}<\varepsilon /2,$$

(15) $$\frac{1}{\beta_{i}}\alpha_{i}<\varepsilon /2,$$

(16) $$\frac{2H}{\left(a_{i}-\frac{\beta_{i}}{\alpha_{i}}\right)}\left[|\beta_{i-m}\left(a_{i}-\frac{\beta_{i}}{\alpha_{i}}\right)-\alpha_{i}b_{i}+L_{i}\alpha_{i}\sum_{j=1}^{m}|c_{(i)j}|+{\bar{L}}_{i}\alpha_{i}\sum_{j=1}^{m}\left|d_{ij}\right|+\alpha_{i}\right]e^{-\left(a_{i}-\frac{\beta_{i}}{\alpha_{i}}\right)\left(a-c\right)}<\varepsilon /4,$$

(17) $$\frac{\xi }{\left(a_{i}-\frac{\beta_{i}}{\alpha_{i}}\right)}\left[|\beta_{i}\left(a_{i}-\frac{\beta_{i}}{\alpha_{i}}\right)-\alpha_{i}b_{i}|+L_{i}\alpha_{i}\sum_{j=1}^{m}|c_{ij}|+{\bar{L}}_{i}\alpha_{i}\sum_{j=1}^{m}\left|d_{ij}\right|+\alpha_{i}\right]<\varepsilon /4.$$

It is true for sufficiently large number *n* that $|\psi_{i}\left(t+t_{n}\right)-\psi_{i}\left(t\right)|<\xi$ and $|h_{i}\left(t+t_{n}\right)-h_{i}\left(t\right)|<\xi$ on [c,b]. Hence, for $\psi \in \Sigma_{0}$, writing

$$\begin{array}{ccc} |\Pi_{i}\psi_{i}\left(t+t_{n}\right)-\Pi_{i}\psi_{i}\left(t\right)| & \leq & \left\{\begin{array}{c} |\frac{1}{\alpha_{i}}\int_{-\infty }^{t}e^{-\frac{\beta_{i}}{\alpha_{i}}\left(t-s\right)}\left(\psi_{i+m}\left(s+t_{n}\right)-\psi_{i+m}\left(s\right)\right)ds|,i=1,\cdots ,m,\\ |\int_{-\infty }^{t}e^{-\left(a_{i-m}-\frac{\beta_{i-m}}{\alpha_{i-m}}\right)\left(t-s\right)}[(\beta_{i-m}\left(a_{i-m}-\frac{\beta_{i-m}}{\alpha_{i-m}}\right)\\ -\alpha_{i-m}b_{i-m})\left(\psi_{i-m}\left(s+t_{n}\right)-\psi_{i-m}\left(s\right)\right)\\ +\alpha_{i-m}\sum_{j=1}^{m}c_{(i-m)j}\left[f_{j}\left(\psi_{j}\left(s+t_{n}\right)\right)-f_{j}\left(\psi_{j}\left(s\right)\right)\right]\\ +\alpha_{i-m}\sum_{j=1}^{m}d_{(i-m)j}\left[g_{j}\left(\psi_{j}\left(\gamma \left(s+t_{n}\right)\right)\right)-g_{j}\left(\psi_{j}\left(\gamma \left(s\right)\right)\right)\right]\\ +\alpha_{i-m}\left(h_{i-m}\left(s+t_{n}\right)-h_{i-m}\left(s\right)\right)ds|,i=m+1,\cdots ,2m. \end{array}\right. \end{array}$$

If we divide the last integral into two parts, we obtain

$$\begin{array}{ccc} |\Pi_{i}\psi_{i}\left(t+t_{n}\right)-\Pi_{i}\psi_{i}\left(t\right)| & \leq & \left\{\begin{array}{c} \left|\frac{1}{\alpha_{i}}\int_{-\infty }^{c}e^{-\frac{\beta_{i}}{\alpha_{i}}\left(t-s\right)}\left(\psi_{i+m}\left(s+t_{n}\right)-\psi_{i+m}\left(s\right)\right)ds\right|\\ +|\frac{1}{\alpha_{i}}\int_{c}^{t}e^{-\frac{\beta_{i}}{\alpha_{i}}\left(t-s\right)}\left(\psi_{i+m}\left(s+t_{n}\right)-\psi_{i+m}\left(s\right)\right)ds|,\;i=1,\cdots ,m,\\ |\int_{-\infty }^{c}e^{-\left(a_{i-m}-\frac{\beta_{i-m}}{\alpha_{i-m}}\right)\left(t-s\right)}[(\beta_{i-m}\left(a_{i-m}-\frac{\beta_{i-m}}{\alpha_{i-m}}\right)\\ -\alpha_{i-m}b_{i-m})\left(\psi_{i-m}\left(s+t_{n}\right)-\psi_{i-m}\left(s\right)\right)\\ +\alpha_{i-m}\sum_{j=1}^{m}c_{(i-m)j}\left[f_{j}\left(\psi_{j}\left(s+t_{n}\right)\right)-f_{j}\left(\psi_{j}\left(s\right)\right)\right]\\ +\alpha_{i-m}\sum_{j=1}^{m}d_{(i-m)j}\left[g_{j}\left(\psi_{j}\left(\gamma \left(s+t_{n}\right)\right)\right)-g_{j}\left(\psi_{j}\left(\gamma \left(s\right)\right)\right)\right]\\ +\alpha_{i-m}\left(h_{i-m}\left(s+t_{n}\right)-h_{i-m}\left(s\right)\right)ds|\\ +|\int_{c}^{t}e^{-\left(a_{i-m}-\frac{\beta_{i-m}}{\alpha_{i-m}}\right)\left(t-s\right)}[(\beta_{i-m}\left(a_{i-m}-\frac{\beta_{i-m}}{\alpha_{i-m}}\right)\\ -\alpha_{i-m}b_{i-m})\left(\psi_{i-m}\left(s+t_{n}\right)-\psi_{i-m}\left(s\right)\right)\\ +\alpha_{i-m}\sum_{j=1}^{m}c_{(i-m)j}\left[f_{j}\left(\psi_{j}\left(s+t_{n}\right)\right)-f_{j}\left(\psi_{j}\left(s\right)\right)\right]\\ +\alpha_{i-m}\sum_{j=1}^{m}d_{(i-m)j}\left[g_{j}\left(\psi_{j}\left(\gamma \left(s+t_{n}\right)\right)\right)-g_{j}\left(\psi_{j}\left(\gamma \left(s\right)\right)\right)\right]\\ +\alpha_{i-m}\left(h_{i-m}\left(s+t_{n}\right)-h_{i-m}\left(s\right)\right)ds|,\;i=m+1,\cdots ,2m, \end{array}\right. \end{array}$$

$$\begin{array}{ccc} \;\;\; & \leq & \left\{\begin{array}{c} \frac{2H}{\beta_{i}}e^{-\frac{\beta_{i}}{\alpha_{i}}\left(a-c\right)}+\frac{1}{\beta_{i}}\alpha_{i},\\ i=1,\cdots ,m,\\ \frac{1}{\left(a_{i-m}-\frac{\beta_{i-m}}{\alpha_{i-m}}\right)}[2H\left|\beta_{i-m}\left(a_{i-m}-\frac{\beta_{i-m}}{\alpha_{i-m}}\right)-\alpha_{i-m}b_{i-m}\right|\\ +2L_{i}H\alpha_{i-m}\sum_{j=1}^{m}|c_{(i-m)j}|+2{\bar{L}}_{i}H\alpha_{i-m}\sum_{j=1}^{m}\left|d_{(i-m)j}\right|\\ +2H\alpha_{i-m}]e^{-(a_{i-m}-\frac{\beta_{i-m}}{\alpha_{i-m}}\left(a-c\right)}\\ +\frac{1}{\left(a_{i-m}-\frac{\beta_{i-m}}{\alpha_{i-m}}\right)}[\left|\beta_{i-m}\left(a_{i-m}-\frac{\beta_{i-m}}{\alpha_{i-m}}\right)-\alpha_{i-m}b_{i-m}\right|\xi \\ +L_{i}\xi \alpha_{i-m}\sum_{j=1}^{m}\left|c_{(i-m)j}\right|+\xi \alpha_{i-m}]\\ +{\bar{L}}_{i}\alpha_{i-m}\sum_{j=1}^{m}d_{(i-m)j}|\int_{c}^{t}e^{-\left(a_{i-m}-\frac{\beta_{i-m}}{\alpha_{i-m}}\right)\left(t-s\right)}\left|\psi_{j}\left(\gamma \left(s+t_{n}\right)\right)-\psi_{j}\left(\gamma \left(s\right)\right)\right|ds,\\ i=m+1,\cdots ,2m. \end{array}\right. \end{array}$$

In the last inequality, we need to evaluate the integral. To do this, let us divide the integral over small intervals as follows. For a fixed t∈[a,b], we assume without loss of generality that $\theta_{i}\leq \theta_{i+l_{n}}-t_{n}$ and $\theta_{i}\leq \theta_{i+l_{n}}-t_{n}=c<\theta_{i+1}<\theta_{i+2}<\cdots <\theta_{i+p}\leq \theta_{i+p+l_{n}}-t_{n}\leq t<\theta_{i+p+1}$. That is, there exist exactly *p* discontinuity points in [c,t].

Let the following inequalities

(18) $$\begin{array}{c} \;\;2{\bar{L}}_{i}\alpha_{i-m}\left(p+1\right)\xi \frac{\left(1-e^{-(a_{i-m}-\frac{\beta_{i-m}}{\alpha_{i-m}})\theta }\right)}{\left(a_{i-m}-\frac{\beta_{i-m}}{\alpha_{i-m}}\right)}<\varepsilon /4, \end{array}$$

and

(19) $$\begin{array}{c} \;\;2{\bar{L}}_{i}\alpha_{i-m}pH\frac{\left(e^{(a_{i-m}-\frac{\beta_{i-m}}{\alpha_{i-m}})\xi }-1\right)}{\left(a_{i-m}-\frac{\beta_{i-m}}{\alpha_{i-m}}\right)}<\varepsilon /4 \end{array}$$

be satisfied for the given ε>0. Let us denote

$$\begin{array}{c} I=\int_{c}^{t}e^{-\left(a_{i-m}-\frac{\beta_{i-m}}{\alpha_{i-m}}\right)\left(t-s\right)}\left|\psi_{j}\left(\gamma \left(s+t_{n}\right)\right)-\psi_{j}\left(\gamma \left(s\right)\right)\right|ds,\;\;i=m+1,\cdots ,2m. \end{array}$$

Consider the last integral as follows:

$$\begin{array}{ccc} \;I & = & \int_{c}^{\theta_{i+1}}e^{-\left(a_{i-m}-\frac{\beta_{i-m}}{\alpha_{i-m}}\right)\left(t-s\right)}\left|\psi_{j}\left(\gamma \left(s+t_{n}\right)\right)-\psi_{j}\left(\gamma \left(s\right)\right)\right|ds\\ & + & \int_{\theta_{i+1}}^{\theta_{i+1+l_{n}}-t_{n}}e^{-\left(a_{i-m}-\frac{\beta_{i-m}}{\alpha_{i-m}}\right)\left(t-s\right)}\left|\psi_{j}\left(\gamma \left(s+t_{n}\right)\right)-\psi_{j}\left(\gamma \left(s\right)\right)\right|ds\\ & + & \int_{\theta_{i+1+l_{n}}-t_{n}}^{\theta_{i+2}}e^{-\left(a_{i-m}-\frac{\beta_{i-m}}{\alpha_{i-m}}\right)\left(t-s\right)}\left|\psi_{j}\left(\gamma \left(s+t_{n}\right)\right)-\psi_{j}\left(\gamma \left(s\right)\right)\right|ds\\ & + & \int_{\theta_{i+2}}^{\theta_{i+2+l_{n}}-t_{n}}e^{-\left(a_{i-m}-\frac{\beta_{i-m}}{\alpha_{i-m}}\right)\left(t-s\right)}\left|\psi_{j}\left(\gamma \left(s+t_{n}\right)\right)-\psi_{j}\left(\gamma \left(s\right)\right)\right|ds\\ & + & \int_{\theta_{i+2+l_{n}}-t_{n}}^{\theta_{i+3}}e^{-\left(a_{i-m}-\frac{\beta_{i-m}}{\alpha_{i-m}}\right)\left(t-s\right)}\left|\psi_{j}\left(\gamma \left(s+t_{n}\right)\right)-\psi_{j}\left(\gamma \left(s\right)\right)\right|ds\\ & \vdots & \\ & + & \int_{\theta_{i+p+l_{n}}-t_{n}}^{t}e^{-\left(a_{i-m}-\frac{\beta_{i-m}}{\alpha_{i-m}}\right)\left(t-s\right)}\left|\psi_{j}\left(\gamma \left(s+t_{n}\right)\right)-\psi_{j}\left(\gamma \left(s\right)\right)\right|ds\\ & = & \sum_{k=i}^{i+p-1}\int_{\theta_{k+l_{n}}-t_{n}}^{\theta_{k+1}}e^{-\left(a_{i-m}-\frac{\beta_{i-m}}{\alpha_{i-m}}\right)\left(t-s\right)}\left|\psi_{j}\left(\gamma \left(s+t_{n}\right)\right)-\psi_{j}\left(\gamma \left(s\right)\right)\right|ds\\ & + & \sum_{k=i}^{i+p-1}\int_{\theta_{k+1}}^{\theta_{k+1+l_{n}}-t_{n}}e^{-\left(a_{i-m}-\frac{\beta_{i-m}}{\alpha_{i-m}}\right)\left(t-s\right)}\left|\psi_{j}\left(\gamma \left(s+t_{n}\right)\right)-\psi_{j}\left(\gamma \left(s\right)\right)\right|ds\\ & + & \int_{\theta_{i+p+l_{n}}-t_{n}}^{t}e^{-\left(a_{i-m}-\frac{\beta_{i-m}}{\alpha_{i-m}}\right)\left(t-s\right)}\left|\psi_{j}\left(\gamma \left(s+t_{n}\right)\right)-\psi_{j}\left(\gamma \left(s\right)\right)\right|ds. \end{array}$$

Denote

$$A_{k}=\int_{\theta_{k+l_{n}}-t_{n}}^{\theta_{k+1}}e^{-\left(a_{i-m}-\frac{\beta_{i-m}}{\alpha_{i-m}}\right)\left(t-s\right)}\left|\psi_{j}\left(\gamma \left(s+t_{n}\right)\right)-\psi_{j}\left(\gamma \left(s\right)\right)\right|ds$$

and

$$B_{k}=\int_{\theta_{k+1}}^{\theta_{k+1+l_{n}}-t_{n}}e^{-\left(a_{i-m}-\frac{\beta_{i-m}}{\alpha_{i-m}}\right)\left(t-s\right)}\left|\psi_{j}\left(\gamma \left(s+t_{n}\right)\right)-\psi_{j}\left(\gamma \left(s\right)\right)\right|ds,$$

where k=i,i+1,⋯,i+p−1, and

$$I=\sum_{k=i}^{i+p-1}A_{k}+\sum_{k=i}^{i+p-1}B_{k}+\int_{\theta_{i+p+l_{n}}-t_{n}}^{t}e^{-\left(a_{i-m}-\frac{\beta_{i-m}}{\alpha_{i-m}}\right)\left(t-s\right)}\left|\psi_{j}\left(\gamma \left(s+t_{n}\right)\right)-\psi_{j}\left(\gamma \left(s\right)\right)\right|ds.$$

By the condition (8) for $t\in [\theta_{k+l_{n}}-t_{n},\theta_{k+1})$, $\gamma \left(t\right)=\xi_{k}$, we have that $\gamma \left(t+t_{n}\right)=\xi_{k+l_{n}}$, k=i,i+1,⋯,i+p−1. Thus, we get that

$$\begin{array}{ccc} A_{k} & = & \int_{\theta_{k+l_{n}}-t_{n}}^{\theta_{k+1}}e^{-\left(a_{i-m}-\frac{\beta_{i-m}}{\alpha_{i-m}}\right)\left(t-s\right)}\left|\psi_{j}\left(\xi_{k+l_{n}}\right)-\psi_{j}\left(\xi_{k}\right)\right|ds\\ & = & \int_{\theta_{k+l_{n}}-t_{n}}^{\theta_{k+1}}e^{-\left(a_{i-m}-\frac{\beta_{i-m}}{\alpha_{i-m}}\right)\left(t-s\right)}\left|\psi_{j}\left(\xi_{k}+t_{n}+o\left(1\right)\right)-\psi_{j}\left(\xi_{k}\right)\right|ds\\ & = & \int_{\theta_{k+l_{n}}-t_{n}}^{\theta_{k+1}}e^{-\left(a_{i-m}-\frac{\beta_{i-m}}{\alpha_{i-m}}\right)\left(t-s\right)}\left|\psi_{j}\left(\xi_{k}+t_{n}\right)-\psi_{j}\left(\xi_{k}\right)+\psi_{j}\left(\xi_{k}+t_{n}+o\left(1\right)\right)-\psi_{j}\left(\xi_{k}+t_{n}\right)\right|ds\;\;\;\\ & \leq & \int_{\theta_{k+l_{n}}-t_{n}}^{\theta_{k+1}}e^{-\left(a_{i-m}-\frac{\beta_{i-m}}{\alpha_{i-m}}\right)\left(t-s\right)}\left[\right|\psi_{j}\left(\xi_{k}+t_{n}\right)-\psi_{j}\left(\xi_{k}\right)\left|+\right|\psi_{j}\left(\xi_{k}+t_{n}+o\left(1\right)\right)-\psi_{j}\left(\xi_{k}+t_{n}\right)\left|\right]ds\\ & \leq & \int_{\theta_{k+l_{n}}-t_{n}}^{\theta_{k+1}}e^{-\left(a_{i-m}-\frac{\beta_{i-m}}{\alpha_{i-m}}\right)\left(t-s\right)}\left[\xi +|\psi_{j}\left(\xi_{k}+t_{n}+o\left(1\right)\right)-\psi_{j}\left(\xi_{k}+t_{n}\right)|\right]ds. \end{array}$$

In accordance with the uniform continuity of ψ, for large *n* and ξ>0, one can define a ρ>0 such that $∥\psi \left(\xi_{k}+t_{n}+o\left(1\right)\right)-\psi \left(\xi_{k}+t_{n}\right)∥<\xi$ if $|\xi_{k+l_{n}}-\xi_{k}-t_{n}|<\rho .$ From this, we deduce that

$$\begin{array}{c} A_{k}\leq 2\xi \int_{\theta_{k-1+l_{n}}-t_{n}}^{\theta_{k}}e^{-\left(a_{i-m}-\frac{\beta_{i-m}}{\alpha_{i-m}}\right)\left(t-s\right)}ds\leq 2\xi \frac{\left(1-e^{-(a_{i-m}-\frac{\beta_{i-m}}{\alpha_{i-m}})\theta }\right)}{\left(a_{i-m}-\frac{\beta_{i-m}}{\alpha_{i-m}}\right)}. \end{array}$$

Moreover, we obtain that

$$\begin{array}{c} B_{k}\leq 2H\int_{\theta_{k}}^{\theta_{k+l_{n}}-t_{n}}e^{-\left(a_{i-m}-\frac{\beta_{i-m}}{\alpha_{i-m}}\right)\left(t-s\right)}ds\leq 2H\frac{\left(e^{(a_{i-m}-\frac{\beta_{i-m}}{\alpha_{i-m}})\xi }-1\right)}{\left(a_{i-m}-\frac{\beta_{i-m}}{\alpha_{i-m}}\right)} \end{array}$$

by virtue of the condition (7). Similarly to $A_{k},$ one can evaluate following integral:

$$\begin{array}{ccc} & & \int_{\theta_{i+p-1+l_{n}}-t_{n}}^{t}e^{-\left(a_{i-m}-\frac{\beta_{i-m}}{\alpha_{i-m}}\right)\left(t-s\right)}|\psi_{j-m}\left(\gamma \left(s+t_{n}\right)\right)-\psi_{j-m}\left(\gamma \left(s\right)\right|ds\\ & & \;\;\;\;\;\;\;\;\;\;\;\;\;\;\;\;\;\;\leq 2\xi \frac{\left(1-e^{-(a_{i-m}-\frac{\beta_{i-m}}{\alpha_{i-m}})\theta }\right)}{\left(a_{i-m}-\frac{\beta_{i-m}}{\alpha_{i-m}}\right)}. \end{array}$$

In this way,

$$\begin{array}{ccc} I & \leq & 2\left(p+1\right)\xi \frac{\left(1-e^{-(a_{i-m}-\frac{\beta_{i-m}}{\alpha_{i-m}})\theta }\right)}{\left(a_{i-m}-\frac{\beta_{i-m}}{\alpha_{i-m}}\right)}+2pH\frac{\left(e^{(a_{i-m}-\frac{\beta_{i-m}}{\alpha_{i-m}})\xi }-1\right)}{\left(a_{i-m}-\frac{\beta_{i-m}}{\alpha_{i-m}}\right)} \end{array}$$

can be obtained. Consequently, it is true that $|\Pi_{i}\psi_{i}\left(t+t_{n}\right)-\Pi_{i}\psi_{i}\left(t\right)|<\varepsilon$ for t∈[a,b] in conformity with the inequalities (14)–(19). This shows that (A2) holds for Πψ. Thus, the operator Π is invariant in $\Sigma_{0}$. □

**Lemma** **5.** *The operator Π from $\Sigma_{0}$ to $\Sigma_{0}$ is contractive.*

**Proof.**  For $u\in \Sigma_{0}$ and $v\in \Sigma_{0}$ and t∈R, one can find that

$$\begin{array}{ccc} |\Pi_{i}u_{i}\left(t\right)-\Pi_{i}v_{i}\left(t\right)| & \leq & \left\{\begin{array}{c} |\frac{1}{\alpha_{i}}\int_{-\infty }^{t}e^{-\frac{\beta_{i}}{\alpha_{i}}\left(t-s\right)}|u_{i+m}\left(s\right)-v_{i+m}\left(s\right)|ds|\leq \frac{1}{\beta_{i}}{∥u\left(t\right)-v\left(t\right)∥}_{1},\\ i=1,\cdots ,m,\\ |\int_{-\infty }^{t}e^{-\left(a_{i-m}-\frac{\beta_{i-m}}{\alpha_{i-m}}\right)\left(t-s\right)}[(\beta_{i-m}\left(a_{i-m}-\frac{\beta_{i-m}}{\alpha_{i-m}}\right)\\ -\alpha_{i-m}b_{i-m})\left(u_{i-m}\left(s\right)-v_{i-m}\left(s\right)\right)\\ +\alpha_{i-m}\sum_{j=1}^{m}c_{(i-m)j}\left(f_{j-m}\left(u_{j-m}\left(s\right)\right)-f_{j-m}\left(v_{j-m}\left(s\right)\right)\right)\\ +\alpha_{i-m}\sum_{j=1}^{m}d_{(i-m)j}\left(g_{j-m}\left(u_{j-m}\left(\gamma \left(s\right)\right)\right)-g_{j-m}\left(v_{j-m}\left(\gamma \left(s\right)\right)\right)\right)ds|\\ \leq \frac{1}{\left(a_{i-m}-\frac{\beta_{i-m}}{\alpha_{i-m}}\right)}[|\beta_{i-m}\left(a_{i-m}-\frac{\beta_{i-m}}{\alpha_{i-m}}\right)-\alpha_{i-m}b_{i-m}\left|\right|\\ +L_{i}\alpha_{i-m}\sum_{j=1}^{m}c_{(i-m)j}+{\bar{L}}_{i}\alpha_{i-m}\sum_{j=1}^{m}d_{(i-m)j}]{∥u\left(t\right)-v\left(t\right)∥}_{1},\\ i=m+1,\cdots ,2m. \end{array}\right. \end{array}$$

So, the inequality ${∥\Pi u-\Pi v∥}_{1}\leq \max_{i}\left(\frac{1}{\beta_{i}},\frac{1}{\left(a_{i}-\frac{\beta_{i}}{\alpha_{i}}\right)}\left[|\beta_{i}\left(a_{i}-\frac{\beta_{i}}{\alpha_{i}}\right)-\alpha_{i}b_{i}|+\alpha_{i}\left(L_{i}\sum_{j=1}^{m}c_{ij}+{\bar{L}}_{i}\sum_{j=1}^{m}d_{ij}\right)\right]\right){∥u-v∥}_{1}$ holds for t∈R.

Consequently, conditions (C3) and (C6) imply that the operator $\Pi :\Sigma_{0}\to \Sigma_{0}$ is contractive. The lemma is proved. □

For convenience, we adopt the following notations:

$$\begin{array}{c} \lambda =\min_{i}\left(\frac{\beta_{i}}{\alpha_{i}},a_{i}-\frac{\beta_{i}}{\alpha_{i}}\right),\;\;L_{f}=\max_{i}\left(\frac{1}{\alpha_{i}},|-\alpha_{i}b_{i}+\beta_{i}\left(a_{i}-\frac{\beta_{i}}{\alpha_{i}}\right)|+\alpha_{i}L_{i}\sum_{j=1}^{m}\left|c_{ij}\right|\right), \end{array}$$

$$\begin{array}{c} L_{g}=\max_{i}\left(\alpha_{i}{\bar{L}}_{i}\sum_{j=1}^{m}\left|d_{ij}\right|\right),\;\;i=1,\cdots ,m. \end{array}$$

The following conditions are to be assumed:

(C7) $\theta [\left(\lambda +L_{f}\right)\left(1+L_{g}\theta \right)e^{(\lambda +L_{f})\theta }+L_{g}]<1$;
(C8) $-\lambda +L_{f}+KL_{g}<0$, where $K={\left(1-\theta [\left(\lambda +L_{f}\right)\left(1+L_{g}\theta \right)e^{(\lambda +L_{f})\theta }+L_{g}]\right)}^{-1}$.

**Theorem** **1.** *If functions $h_{i}\left(t\right)$, i=1,2,…m, in system (4), are Poisson stable with the convergence sequence $t_{n},n=1,2,\ldots$, then under conditions (C1)–(C8), system (4) has a unique exponentially stable Poisson stable solution.*

**Proof.**  First, we show the completeness of the space $\Sigma_{0}$. Let us denote a Cauchy sequence in the space $\Sigma_{0}$ by $r_{k}\left(t\right),$ where the limit of $r_{k}\left(t\right)$, on R as k→∞ is r(t). One can say that r(t) is a bounded function, which means that (A2) is achieved for r(t). Let us show that (A1) is satisfied for r(t) as well. Consider a closed, bounded interval I⊂R. We obtain

$$\begin{array}{ccc} ∥r\left(t+t_{n}\right)-r\left(t\right)∥ & \leq & ∥r\left(t+t_{n}\right)-r_{k}\left(t+t_{n}\right)∥\\ & + & ∥r_{k}\left(t+t_{n}\right)-r_{k}\left(t\right)∥+∥r_{k}\left(t\right)-r\left(t\right)∥. \end{array}$$

For small enough ε>0 and t∈I, each difference in absolute value on the right side of last the inequality can be made smaller than $\frac{\varepsilon }{3}$, and then we have $∥r\left(t+t_{n}\right)-r\left(t\right)∥<\varepsilon$ on I. This implies that the sequence $r\left(t+t_{n}\right)\to r\left(t\right)$ uniformly on *I*, which approves that the space $\Sigma_{0}$ is complete. Note that the operator Π is invariant and contractive in $\Sigma_{0}$, on the ground of Lemmas 4 and 5, respectively. It follows from the Banach theorem that the operator Π has only one fixed point $z\left(t\right)\in \Sigma_{0}$. That is, we concluded that the system (4) has a unique solution. So, the uniqueness of the solution is shown.

Next, consider the stability of z(t). Further, for convenience, write the system (11) in vector form, using the 2m-dimensional function $z\left(t\right)=(u_{1}\left(t\right),\ldots ,u_{m}\left(t\right),y_{1}\left(t\right),\ldots ,y_{m}\left(t\right))$,

(20) $$\frac{dz}{dt}=Az+F\left(t,z\right),$$

where $A=\left\{-\frac{\beta_{1}}{\alpha_{1}},-\frac{\beta_{2}}{\alpha_{2}},\cdots ,-\frac{\beta_{m}}{\alpha_{m}},-\left(a_{1}-\frac{\beta_{1}}{\alpha_{1}}\right),-\left(a_{2}-\frac{\beta_{2}}{\alpha_{2}}\right),\cdots ,-\left(a_{m}-\frac{\beta_{m}}{\alpha_{m}}\right)\right\}$ is a diagonal matrix, $F\left(t,z\right)=(F_{1}\left(t,z\right),F_{2}\left(t,z\right),\cdots ,F_{2m}\left(t,z\right))$ is a vector function such that

$$\begin{array}{ccc} F_{i}\left(t,z\right) & = & \left\{\begin{array}{c} \frac{1}{\alpha_{i}}z_{i+m}\left(t\right),\;i=1,\cdots ,m\\ -\left(\alpha_{i-m}b_{i-m}-\beta_{i-m}\left(a_{i-m}-\frac{\beta_{i-m}}{\alpha_{i-m}}\right)\right)z_{i-m}\left(t\right)\\ +\alpha_{i-m}\sum_{j=1}^{m}c_{(i-m)j}f_{j}\left(z_{j}\left(t\right)\right)+\alpha_{i-m}\sum_{j=1}^{m}d_{(i-m)j}g_{j}\left(z_{j}\left(\gamma \left(t\right)\right)\right)+\alpha_{i-m}h_{i-m}\left(t\right),\\ i=m+1,\cdots ,2m. \end{array}\right. \end{array}$$

The exponential stability will be proved once we prove the lemma below.

**Lemma** **6.** *Suppose that conditions (C1) and (C8) hold, and z(t) is a continuous and bounded function with ∥z(t)∥<H. If $\omega \left(t\right)=(u_{1}\left(t\right),..,u_{m}\left(t\right),v_{1}\left(t\right),\cdots ,v_{m}\left(t\right))$ is a solution of*

(21) $$\omega^{\prime }\left(t\right)=A\omega \left(t\right)+F\left(t,\omega \left(t\right)\right),$$

*where $A=\left\{-\frac{\beta_{1}}{\alpha_{1}},-\frac{\beta_{2}}{\alpha_{2}},\cdots ,-\frac{\beta_{m}}{\alpha_{m}},-\left(a_{1}-\frac{\beta_{1}}{\alpha_{1}}\right),-\left(a_{2}-\frac{\beta_{2}}{\alpha_{2}}\right),\cdots ,-\left(a_{m}-\frac{\beta_{m}}{\alpha_{m}}\right)\right\}$ is a diagonal matrix, $F\left(t,\omega \right)=(F_{1}\left(t,\omega \right),F_{2}\left(t,\omega \right),\cdots ,F_{2m}\left(t,\omega \right))$ is a vector function such that*

$$\begin{array}{ccc} F_{i}\left(t,\omega \left(t\right)\right) & = & \left\{\begin{array}{c} \frac{1}{\alpha_{i}}\omega_{i+m}\left(t\right),\;i=1,\cdots ,m,\\ -\left(\alpha_{i-m}b_{i-m}-\beta_{i-m}\left(a_{i-m}-\frac{\beta_{i-m}}{\alpha_{i-m}}\right)\right)\omega_{i-m}\left(t\right)\\ +\alpha_{i-m}\sum_{j=1}^{m}c_{(i-m)j}\left[f_{j}\left(\omega_{j}\left(t\right)+z_{j}\left(t\right)\right)-f_{j}\left(z_{j}\left(t\right)\right)\right]\\ +\alpha_{i-m}\sum_{j=1}^{m}d_{(i-m)j}\left[g_{j}\left(\omega_{j}\left(\gamma \left(t\right)\right)+z_{j}\left(\gamma \left(t\right)\right)\right)-g_{j}\left(z_{j}\left(\gamma \left(t\right)\right)\right)\right],\\ i=m+1,\cdots ,2m, \end{array}\right. \end{array}$$

*and then the following inequality*

(22) $$\begin{array}{c} \;\;\;\;\;∥w(\gamma (t))∥\leq K∥w(t)∥ \end{array}$$

*is true for t∈R, with K, which is defined as in (C8).*

**Proof.**  Let *t* belong to the interval $\left[\theta_{k},\theta_{k+1}\right)$, for some fixed *k*, and consider two alternative cases (a) $\theta_{k}\leq \xi_{k}\leq t<\theta_{k+1}$, (b) $\theta_{k}\leq t<\xi_{k}<\theta_{k+1}$.

(a) For $t\geq \xi_{k}$, we have

$$\begin{array}{ccc} \left||w(t)|\right| & \leq & ||w\left(\xi_{k}\right)\left|\right|+\int_{\xi_{k}}^{t}\left[||A||||w(s)||+||F(s,\omega (s))||\right]ds\\ & \leq & ||w\left(\xi_{k}\right)\left|\right|+\int_{\xi_{k}}^{t}\left[\lambda ||w\left(s\right)||+L_{f}\left||w\left(s\right)|\right|+L_{g}||w\left(\xi_{k}\right)\left|\right|\right]ds\\ & \leq & ||w\left(\xi_{k}\right)\left|\right|\left(1+L_{g}\theta \right)+\int_{\xi_{i}}^{t}\left(\lambda +L_{f}\right)||w\left(s\right)\left|\right|ds. \end{array}$$

According to the Gronwall–Bellman lemma, we obtain

$$\begin{array}{c} \;\;\;||w\left(t\right)||\leq ||w\left(\xi_{k}\right)\left|\right|\left(1+L_{g}\theta \right)e^{(\lambda +L_{f})\theta }. \end{array}$$

Moreover, for $t\in \left[\theta_{k},\theta_{k+1}\right)$ we have

$$\begin{array}{ccc} \left||w(\xi_{k})|\right| & \leq & \left||w\left(t\right)|\right|+\int_{\xi_{k}}^{t}\left[||A||||w(s)||+||F(s,\omega (s))||\right]ds\\ & \leq & \left||w\left(t\right)|\right|+\int_{\xi_{k}}^{t}[\left(\lambda +L_{f}\right)||w\left(s\right)\left|\right|+L_{g}||w\left(\xi_{k}\right)||]ds\\ & \leq & \left||w\left(t\right)|\right|+\int_{\xi_{k}}^{t}\left[\left(\lambda +L_{f}\right)\left(1+L_{g}\theta \right)e^{(\lambda +L_{f})\theta }||w\left(\xi_{k}\right)\left|\right|+L_{g}||w\left(\xi_{k}\right)\left|\right|\right]ds\\ & \leq & \left||w\left(t\right)|\right|+\theta \left[\left(\lambda +L_{f}\right)\left(1+L_{g}\theta \right)e^{(\lambda +L_{f})\theta }+L_{g}\right]||w\left(\xi_{k}\right)\left|\right|. \end{array}$$

Deduce from the condition (C8) that $∥w\left(\xi_{k}\right)∥\leq K∥w\left(t\right)∥$, for $t\in \left[\theta_{k},\theta_{k+1}\right),k\in Z$. It follows that (22) holds for all $\theta_{k}\leq \xi_{k}\leq t<\theta_{k+1}$, k∈Z. If one considers another case, $\theta_{k}\leq t<\xi_{k}<\theta_{k+1},k\in Z$; in a similar way, we will obtain the same result. Thus, (22) is satisfied for all t∈R. □

Let ω(t)=υ(t)−z(t), where $\upsilon \left(t\right)=colon(\upsilon_{1}\left(t\right),\upsilon_{2}\left(t\right),\cdots ,\upsilon_{m}\left(t\right))$ denotes any other solution of the system (4). We will check that $\omega \left(t\right)=colon(\omega_{1}\left(t\right),\omega_{2}\left(t\right),\cdots ,\omega_{m}\left(t\right))$ is a solution of (21).

Hence, the inequality

(23) $$\begin{array}{c} \;||w\left(t\right)||\leq e^{A(t-t_{0})}∥w\left(t_{0}\right)∥+\int_{t_{0}}^{t}e^{A(t-s)}F\left(s,\omega \left(s\right)\right)ds. \end{array}$$

is valid. By using the inequality (22) to (23), we have that

$$\begin{array}{ccc} \left||w(t)|\right| & \leq & e^{-\lambda (t-t_{0})}||w\left(t_{0}\right)\left|\right|+\int_{t_{0}}^{t}e^{-\lambda (t-s)}\left[L_{f}\;||w\left(s\right)||+L_{g}K∥w\left(s\right)∥\right]ds. \end{array}$$

Hence, we find that

$$\begin{array}{c} \;∥w\left(t\right)∥\leq e^{-\lambda (t-t_{0})}∥w\left(t_{0}\right)∥+\int_{t_{0}}^{t}e^{-\lambda (t-s)}\left(L_{f}+KL_{g}\right)||w\left(s\right)\left|\right|ds. \end{array}$$

It means that

$$\begin{array}{c} \;e^{\lambda t}∥w\left(t\right)∥\leq e^{\lambda t_{0}}||w\left(t_{0}\right)\left|\right|+\left(L_{f}+KL_{g}\right)\int_{t_{0}}^{t}e^{\lambda s}∥w\left(s\right)∥ds. \end{array}$$

By the Gronwall–Bellman lemma, the following inequality can be obtained:

$$\begin{array}{c} \;\;\;\;||w\left(t\right)||\leq ||w\left(t_{0}\right)\left|\right|e^{\left(-\lambda +L_{f}+KL_{g}\right)\left(t-t_{0}\right)}. \end{array}$$

In other words, we have

$$\begin{array}{c} \;\;\;||\upsilon \left(t\right)-z\left(t\right)||\leq ||\upsilon \left(t_{0}\right)-z\left(t_{0}\right)\left|\right|e^{\left(-\lambda +L_{f}+KL_{g}\right)\left(t-t_{0}\right)}. \end{array}$$

It follows from the condition (C7), that Poisson stable solution z(t) of (4) is exponentially stable. The theorem is proved.

Next, we shall need the following condition:

(C9) The functions $h_{i}\left(t\right)$, i=1,2,…m, in system (4) belong to $\Sigma_{0}$, and there exist positive numbers $\epsilon_{0},\delta$ and sequence $s_{n},n=1,2,\ldots ,$ which diverge to infinity such that $∥h\left(t+t_{n}\right)-h\left(t\right)∥\geq \epsilon_{0}$ for each $t\in [s_{n}-\delta ,s_{n}+\delta ]$ and n∈N.

The unpredictability of the solution for the system (4) is established by the next theorem.

**Theorem** **2.** *Under conditions (C1)–(C9), system (4) has a unique exponentially stable unpredictable solution.*

According to Theorem 1, system (4) has a unique exponentially stable Poisson stable solution z(t). Therefore, to show that system (4) has a unique exponentially stable unpredictable solution, it remains to prove that z(t) admits the unpredictability property.

So, we will show the existence of a sequence $s_{n}$, satisfying $l_{n}\to \infty$, as n→∞, and numbers $\epsilon_{0}>0,\delta >0$ so that $|z_{i}\left(t+t_{n}\right)-z_{i}\left(t\right)|\geq \epsilon_{0}$ for each $t\in [s_{n}-\delta ,s_{n}+\delta ]$ and n∈N

(24) $$\begin{array}{c} z_{i}\left(t+t_{n}\right)-z_{i}\left(t\right)=\left\{\begin{array}{c} z_{i}\left(s_{n}+t_{n}\right)-z_{i}\left(s_{n}\right)-\frac{\beta_{i}}{\alpha_{i}}\int_{s_{n}}^{t}\left(z_{i}\left(s+t_{n}\right)-z_{i}\left(s\right)\right)ds\\ +\int_{s_{n}}^{t}\frac{1}{\alpha_{i}}\left(z_{i+m}\left(s+t_{n}\right)-z_{i+m}\left(s\right)\right)ds,\;i=1,\cdots ,m,\\ z_{i}\left(s_{n}+t_{n}\right)-z_{i}\left(s_{n}\right)-\int_{s_{n}}^{t}\left(a_{i-m}-\frac{\beta_{i-m}}{\alpha_{i-m}}\right)\left(z_{i}\left(s+t_{n}\right)-z_{i}\left(s\right)\right)ds\\ -\int_{s_{n}}^{t}\left(\beta_{i-m}\left(a_{i-m}-\frac{\beta_{i-m}}{\alpha_{i-m}}\right)-\alpha_{i-m}b_{i-m}\right)\left(z_{i-m}\left(s+t_{n}\right)-z_{i-m}\left(s\right)\right)ds\\ +\int_{s_{n}}^{t}\alpha_{i-m}\sum_{j=1}^{m}c_{(i-m)j}\left[f_{j}\left(z_{j}\left(s+t_{n}\right)\right)-f_{j}\left(z_{j}\left(s\right)\right)\right]ds\\ +\int_{s_{n}}^{t}\alpha_{i-m}\sum_{j=1}^{m}d_{(i-m)j}\left[g_{j}\left(z_{j}\left(\gamma \left(s+t_{n}\right)\right)\right)-g_{j}\left(z_{j}\left(\gamma \left(s\right)\right)\right)\right]ds\\ +\int_{s_{n}}^{t}\alpha_{i-m}\left(h_{i-m}\left(s+t_{n}\right)-h_{i-m}\left(s\right)\right)ds,i=m+1,m+2,\cdots ,2m. \end{array}\right. \end{array}$$

Positive numbers κ and l,k∈N are chosen to satisfy

(25) κ<δ,

(26) $$\kappa \left[\frac{\alpha_{i}}{2}-\left[\left(a_{i}-\frac{\beta_{i}}{\alpha_{i}}\right)+|\beta_{i}\left(a_{i}-\frac{\beta_{i}}{\alpha_{i}}\right)-\alpha_{i}b_{i}|+L_{i}\alpha_{i}\sum_{j=1}^{m}\left|c_{ij}\right|\right]\left(\frac{1}{l}+\frac{2}{k}\right)-2{\bar{L}}_{i}\alpha_{i}\sum_{j=1}^{m}\left|d_{ij}\right|\right]\geq 3/2l,\;i=1,\cdots ,m$$

and

(27) $$|z_{i}\left(t+s\right)-z_{i}\left(t\right)|<\epsilon_{0}\min\left\{1/k,1/4l\right\},\;t\in R,\;\left|s\right|<\kappa ,\;i=1,\cdots ,m.$$

Let the numbers κ,l,k and n∈N be fixed.

We will use the symbol Δ to denote $|z_{i}\left(s_{n}+t_{n}\right)-z_{i}\left(t_{n}\right)|$ and examine the cases: $\left(i\right)\;\Delta \geq \epsilon_{0}/l,$$\left(ii\right)\;\Delta <\epsilon_{0}/l.$

(i) If $\Delta \geq \epsilon_{0}/l$ is valid, then

(28) $$\begin{array}{ccc} |z_{i}\left(t+s\right)-z_{i}\left(t\right)| & \geq & |z_{i}\left(s_{n}+t_{n}\right)-z_{i}\left(s_{n}\right)\left|-\right|z_{i}\left(s_{n}\right)-z_{i}\left(t\right)|\\ & - & |z_{i}\left(t+t_{n}\right)-z_{i}\left(s_{n}+t_{n}\right)|\\ & > & \epsilon_{0}/l-\epsilon_{0}/4l-\epsilon_{0}/4l=\epsilon_{0}/2l,i=m+1,m+2,\cdots ,2m \end{array}$$

for $t\in [s_{n}-\kappa ,s_{n}+\kappa ],$n∈N.

(ii) For the case $\Delta <\epsilon_{0}/l$, in accordance with (27), we have that

(29) $$\begin{array}{ccc} |z_{i}\left(t+t_{n}\right)-z_{i}\left(t\right)| & \leq & |z_{i}\left(s_{n}+t_{n}\right)-z_{i}\left(s_{n}\right)\left|+\right|z_{i}\left(s_{n}\right)-z_{i}\left(t\right)|\\ & + & |z_{i}\left(t+t_{n}\right)-z_{i}\left(s_{n}+t_{n}\right)|\\ & < & \epsilon_{0}/l+\epsilon_{0}/k+\epsilon_{0}/k=\left(1/l+2/k\right)\epsilon_{0},\;i=1,2,\cdots ,2m, \end{array}$$

for $t\in [s_{n},s_{n}+\kappa ].$

Applying (25)–(27) and due to the condition (C9), one can find that

$$\begin{array}{ccc} |z_{i}\left(t+t_{n}\right)-z_{i}\left(t\right)| & \geq & \left|\int_{s_{n}}^{t}\alpha_{i-m}\left(h_{i-m}\left(s+t_{n}\right)-h_{i-m}\left(s\right)\right)ds\right|\\ & - & \left|\int_{s_{n}}^{t}\left(a_{i-m}-\frac{\beta_{i-m}}{\alpha_{i-m}}\right)\left(z_{i}\left(s+t_{n}\right)-z_{i}\left(s\right)\right)ds\right|\\ & - & \left|\int_{s_{n}}^{t}\left(\beta_{i-m}\left(a_{i-m}-\frac{\beta_{i-m}}{\alpha_{i-m}}\right)-\alpha_{i-m}b_{i-m}\right)\left(z_{i-m}\left(s+t_{n}\right)-z_{i-m}\left(s\right)\right)ds\right|\\ & - & \left|\int_{s_{n}}^{t}\alpha_{i-m}\sum_{j=1}^{m}c_{(i-m)j}\left[f_{j}\left(z_{j}\left(s+t_{n}\right)\right)-f_{j}\left(z_{j}\left(s\right)\right)\right]ds\right|\\ & - & \left|\int_{s_{n}}^{t}\alpha_{i-m}\sum_{j=1}^{m}d_{(i-m)j}\left[g_{j}\left(z_{j}\left(\gamma \left(s+t_{n}\right)\right)\right)-g_{j}\left(z_{j}\left(\gamma \left(s\right)\right)\right)\right]ds\right|\\ & - & |z_{i}\left(s_{n}+t_{n}\right)-z_{i}\left(s_{n}\right)|\\ & \geq & \alpha_{i-m}\epsilon_{0}\frac{\kappa }{2}-\epsilon_{0}\left(\frac{1}{l}+\frac{2}{k}\right)\kappa [\left(a_{i-m}-\frac{\beta_{i-m}}{\alpha_{i-m}}\right)\\ & + & |\beta_{i-m}\left(a_{i-m}-\frac{\beta_{i-m}}{\alpha_{i-m}}\right)-\alpha_{i-m}b_{i-m}|+L_{i}\alpha_{i-m}\sum_{j=1}^{m}\left|c_{(i-m)j}\right|]\\ & - & {\bar{L}}_{i}\alpha_{i-m}\sum_{j=1}^{m}|d_{(i-m)j}\left|\right|\int_{s_{n}}^{t}\left|z_{j}\left(\gamma \left(s+t_{n}\right)\right)-z_{j}\left(\gamma \left(s\right)\right)\right|ds]-\frac{\epsilon_{0}}{l} \end{array}$$

for $t\in [s_{n}+\frac{\kappa }{2},s_{n}+\kappa ]$, i=m+1,m+2,⋯,2m.

For a fixed $t\in [s_{n}+\frac{\kappa }{2},s_{n}+\kappa ],$ we can choose κ to be small enough so that $\theta_{i+l_{n}}-t_{n}\leq s_{n}<s_{n}+\frac{\kappa }{2}\leq t\leq s_{n}+\kappa <\theta_{i+1}$ for certain i∈Z. The function z(t) is uniformly continuous since it belongs to $\Sigma_{0}$. We can conclude that for sufficiently large *n* and $\epsilon_{0}>0$, there exists number ρ>0 such that

$$\begin{array}{ccc} \int_{s_{n}}^{t}\left|z_{j}\left(\gamma \left(s+t_{n}\right)\right)-z_{j}\left(\gamma \left(s\right)\right)\right|ds & = & \int_{s_{n}}^{t}\left|z_{j}\left(\xi_{i+l_{n}}\right)-z_{j}\left(\xi_{i}\right)\right|ds\\ & \leq & \int_{s_{n}}^{t}\left|z_{j}\left(\xi_{i}+t_{n}\right)-z_{i}\left(\xi_{i}\right)\right|ds\\ & + & \int_{s_{n}}^{t}\left|z_{j}\left(\xi_{i}+t_{n}+o\left(1\right)\right)-z_{j}\left(\xi_{i}+t_{n}\right)\right|ds\leq 2\kappa \epsilon_{0}, \end{array}$$

if $|\xi_{i+l_{n}}-\xi_{i}-t_{n}|<\rho .$

Finally, we have by inequalities (25)–(27) that

$$\begin{array}{ccc} |z_{j}\left(t+t_{n}\right)-z_{j}\left(t\right)| & \geq & \alpha_{i-m}\epsilon_{0}\frac{\kappa }{2}-\epsilon_{0}\left(\frac{1}{l}+\frac{2}{k}\right)\kappa [\left(a_{i-m}-\frac{\beta_{i-m}}{\alpha_{i-m}}\right)\\ & + & |\beta_{i-m}\left(a_{i-m}-\frac{\beta_{i-m}}{\alpha_{i-m}}\right)-\alpha_{i-m}b_{i-m}|+L_{i}\alpha_{i-m}\sum_{j=1}^{m}\left|c_{(i-m)j}\right|]\\ & - & 2{\bar{L}}_{i}\alpha_{i-m}\sum_{j=1}^{m}\left|d_{(i-m)j}\right|\kappa \epsilon_{0}-\frac{\epsilon_{0}}{l}\geq -\frac{\epsilon_{0}}{l}+\frac{3\epsilon_{0}}{2l}\geq \frac{\epsilon_{0}}{2l} \end{array}$$

for $t\in [s_{n}+\frac{\kappa }{2},s_{n}+\kappa ]$, i=m+1,m+2,⋯,2m. Thus, we obtain that

$$\begin{array}{c} |z_{j}\left(t+t_{n}\right)-z_{j}\left(t\right)|\geq \frac{\epsilon_{0}}{2l} \end{array}$$

for $t\in [s_{n}+\frac{\kappa }{2},s_{n}+\kappa ]$, i=m+1,m+2,⋯,2m. Moreover,

$$\begin{array}{ccc} |z_{i}\left(t+t_{n}\right)-z_{i}\left(t\right)| & \geq & \left|\int_{s_{n}}^{t}\frac{1}{\alpha_{i}}\left(z_{i+m}\left(s+t_{n}\right)-z_{i+m}\left(s\right)\right)ds\right|\\ & - & |z_{i}\left(s_{n}+t_{n}\right)-z_{i}\left(s_{n}\right)|\\ & - & \left|\frac{\beta_{i}}{\alpha_{i}}\int_{s_{n}}^{t}\left(z_{i}\left(s+t_{n}\right)-z_{i}\left(s\right)\right)ds\right|\\ & \geq & \frac{1}{\alpha_{i}}\kappa \frac{\epsilon_{0}}{2l}-\frac{\epsilon_{0}}{l}-\frac{\beta_{i}}{\alpha_{i}}\kappa \epsilon_{0}\left(\frac{1}{l}+\frac{2}{k}\right). \end{array}$$

Thus, z(t) is an unpredictable solution with sequences ${\bar{s}}_{n}=s_{n}+\frac{3\kappa }{4}$ and ${\bar{\delta }}_{n}=\frac{\kappa }{4}$.

## 4. An Example and Numerical Simulations

Consider the following inertial neural network with generalized piecewise constant argument given by

(30) $$u_{i}^{\prime \prime }\left(t\right)=-a_{i}u_{i}^{\prime }\left(t\right)-b_{i}u_{i}\left(t\right)+\sum_{j=1}^{3}c_{ij}f_{j}\left(u_{j}\left(t\right)\right)+\sum_{j=1}^{3}d_{ij}g_{j}\left(u_{j}\left(\gamma \left(t\right)\right)\right)+h_{i}\left(t\right),$$

where the damping coefficients and the rates of the neurons activity are given as follows: $a_{1}=a_{2}=4,a_{3}=6,b_{1}=b_{2}=3.8,b_{3}=6.2$. As activations, we consider the following functions $f_{i}\left(u_{i}\left(t\right)\right)=0.2\tanh\left(u_{i}\left(t\right)/4\right)$, $g_{i}\left(u_{i}\left(t\right)\right)=0.3\tanh\left(u_{i}\left(t\right)/6\right),$i=1,2,3, and the synaptic connection weights are given by

$$\left(\begin{array}{ccc} c_{11} & c_{12} & c_{13}\\ c_{21} & c_{22} & c_{23}\\ c_{31} & c_{32} & c_{33} \end{array}\right)=\left(\begin{array}{ccc} 0.1 & \;0.2 & \;0.5\\ 0.3 & \;0.1 & \;0.2\\ 0.2 & \;0.1 & \;0.3 \end{array}\right),\;\;\;\left(\begin{array}{ccc} d_{11} & d_{12} & d_{13}\\ d_{21} & d_{22} & d_{23}\\ d_{31} & d_{32} & d_{33} \end{array}\right)=\left(\begin{array}{ccc} 0.1 & \;0.2 & \;0.1\\ 0.2 & \;0.2 & \;0.2\\ 0.1 & \;0.3 & \;0.1 \end{array}\right)$$

and the external input

$$\left(\begin{array}{c} h_{1}\left(t\right)\\ h_{2}\left(t\right)\\ h_{3}\left(t\right) \end{array}\right)=\left(\begin{array}{c} -4.8\Theta (t)\\ -36\Theta^{3}\left(t\right)+0.4\\ 32\Theta^{3}\left(t\right)-0.3 \end{array}\right).$$

As the function Θ(t), we use an unpredictable function,

$$\Theta \left(t\right)=\int_{-\infty }^{t}e^{-3(t-s)}\Omega \left(s\right)ds,\;t\in R,$$

where Ω(t) is piecewise constant function, which is defined by $\Omega \left(t\right)=\pi_{i}$ for t∈[i,i+1),i∈Z with an unpredictable solution $\pi_{i},\;i\in Z$, of logistic map considered in the paper [26]. The function Θ(t) is bounded on R such that $\underset{t\in R}{\sup}\left|\Theta (t)\right|\leq 1/3.$ In the paper [26], it was proved that the function Θ(t) is unpredictable.

The constant argument function γ(t) is defined by the sequences $\theta_{k}=\frac{k}{5}$, $\xi_{k}=\frac{2k+1}{10}$, k∈Z, which constitute the Poisson triple (see example, Preliminaries).

We checked that the conditions (C1)–(C9) are true for the system (30) with $\alpha_{1}=\alpha_{2}=\alpha_{3}=2,\beta_{1}=\beta_{2}=3,\;\beta_{3}=4$, λ=1.3,$m_{f}=0.2,\;m_{g}=0.3$, $L_{i}={\bar{L}}_{i}=0.05,$ for i=1,2,3, and moreover, K=2.164. If we take H=2, then (30) satisfies all conditions of Theorem 2.

Since we will not be able to build the unpredictable function itself, we will not be able to accurately determine the initial value. Then, to show the behavior of an unpredictable solution u(t), according to the stability, one can consider another solution $z\left(t\right)=(z_{1}\left(t\right),z_{2}\left(t\right),z_{3}\left(t\right))$, which exponentially approaches this solution and starts at the initial point z(0)=(0.4956,1.7739,1.1992).

**Figure 1:**
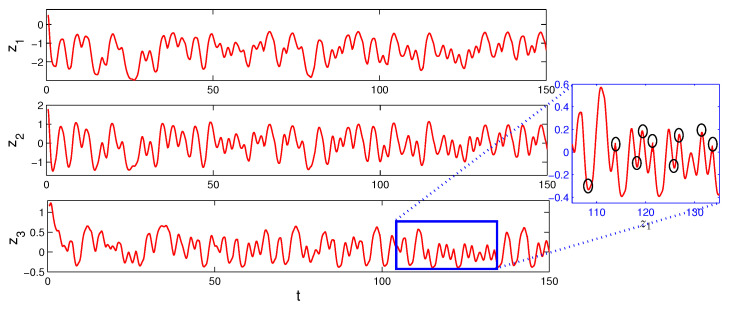
The coordinates of function z(t) exponentially approaches the unpredictable solution u(t). For the special part $z_{3}$ between 105 and 140, it is zoomed to demonstrate the appearance of non-smooth or discontinuous derivatives, with $\Omega \left(t\right)=\pi_{i}$ for t∈[i,i+1), i∈Z.

**Figure 2:**
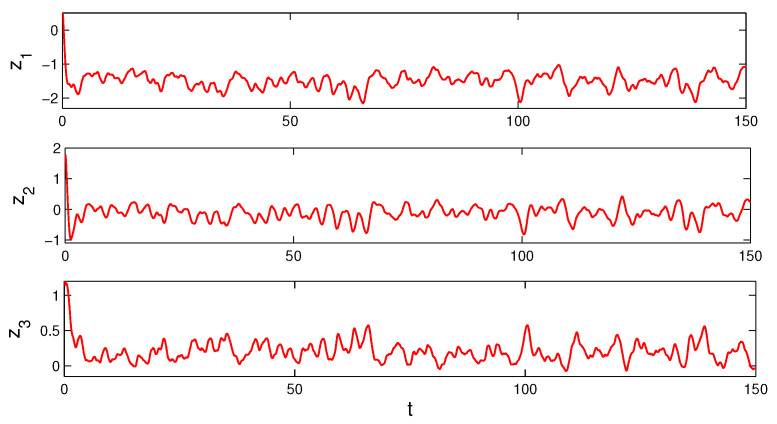
The coordinates of function z(t), with $\Omega \left(t\right)=\pi_{i}$ for t∈[0.1i,0.1(i+1)), i∈Z.

**Figure 3:**
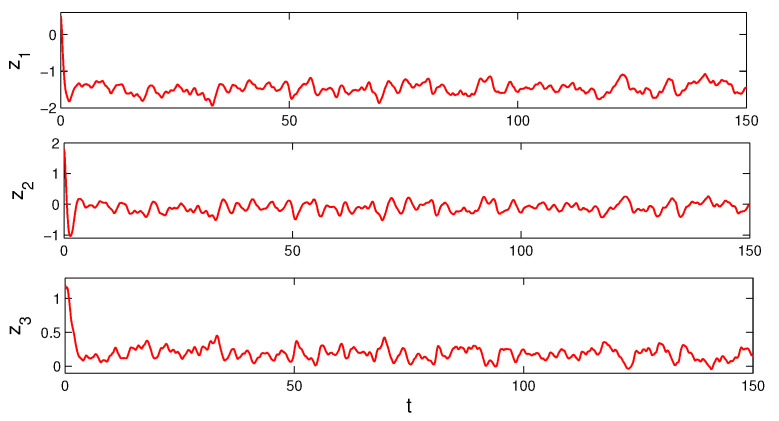
The coordinates of function z(t), when $\Omega \left(t\right)=\pi_{i}$ for t∈[0.05i,0.05(i+1)), i∈Z.

**Figure 4:**
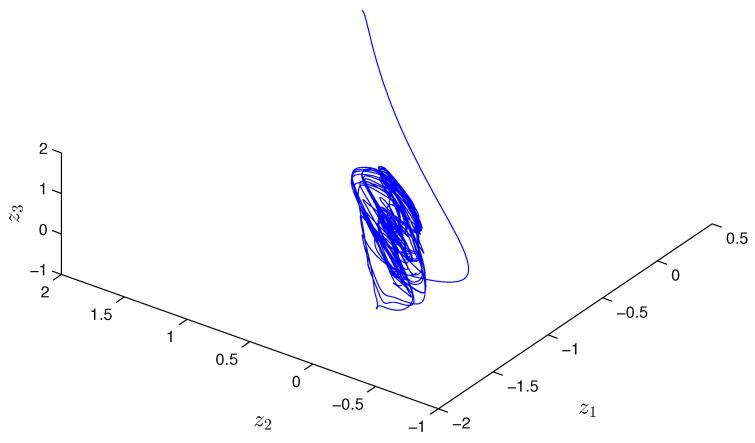
The trajectory of function z(t).

As can be seen in Figure 1, the solution of the system (30) is a continuous function. However, due to the constant argument in the model, a function with discontinuous derivatives of the first order is obtained, and we have continuously differentiable motion on the intervals $[\theta_{k},\theta_{k+1}),k\in Z$. That is, we have the non-smoothness at the switching points $\theta_{k},k\in Z$. Next, let us observe the influence of the length of constancy in the delay function for the output dynamics. Namely, we additionally construct the function $\Omega \left(t\right)=\pi_{i}$ in the intervals [0.1i,0.1(i+1)) and [0.05i,0.05(i+1))i∈Z. The result of the simulations is seen in Figure 2 and Figure 3, where the intensity of the non-smoothness is increased, if one compares with Figure 1, where the length of the constancy is equal to 1. Figure 4 demonstrates the chaotic nature of the unpredictability. Furthermore, this picture confirms the existence of an attractor.

## 5. Conclusions

The article discusses Poisson stable and irregular, that is, unpredictable, motions in inertial neural networks with a generalized piecewise constant argument. The novelty of the research is caused by the fact that the neural network model contains a discontinuous Poisson stable function as a piecewise constant argument. The proof of the convergence of discontinuous functions was made on the basis of the B-topology, and the included intervals method was used. Since the argument function is defined by time sequences, they led to difficulties in the study, respectively. To solve this problem, we needed the concepts of a Poisson couple and a Poisson triple of sequences.

The research technique and results can be effectively used in the study of different models of neural networks with impulses and discontinuous functional dependence on time, as well as areas of their application.

## Data Availability

Not applicable.
